# *Lactococcus lactis* secreting phage lysins as a potential antimicrobial against multi-drug resistant *Staphylococcus aureus*

**DOI:** 10.7717/peerj.12648

**Published:** 2022-03-01

**Authors:** Carumathy Chandran, Hong Yun Tham, Raha Abdul Rahim, Swee Hua Erin Lim, Khatijah Yusoff, Adelene Ai-Lian Song

**Affiliations:** 1 Department of Microbiology, Universiti Putra Malaysia, Serdang, Selangor, Malaysia; 2 Department of Cell and Molecular Biology, Universiti Putra Malaysia, Serdang, Selangor, Malaysia; 3 Institute of Bioscience, Universiti Putra Malaysia, Serdang, Selangor, Malaysia; 4 Health Science Division, Abu Dhabi Women’s College, Abu Dhabi, United Arab Emirates

**Keywords:** Endolysin, Bacteriophage, Multi-drug resistant Staphylococcus aureus, Virion associated peptidoglycan hydrolase, *Lactococcus lactis*

## Abstract

**Background:**

*Staphylococcus aureus* is an opportunistic Gram-positive bacterium that can form biofilm and become resistant to many types of antibiotics. The treatment of multi-drug resistant *Staphylococcus aureus* (MDRSA) infection is difficult since it possesses multiple antibiotic-resistant mechanisms. Endolysin and virion-associated peptidoglycan hydrolases (VAPGH) enzymes from bacteriophage have been identified as potential alternative antimicrobial agents. This study aimed to assess the ability of *Lactococcus lactis* NZ9000 secreting endolysin and VAPGH from *S. aureus* bacteriophage 88 to inhibit the growth of *S. aureus* PS 88, a MDRSA.

**Method:**

Endolysin and VAPGH genes were cloned and expressed in *L. lactis* NZ9000 after fusion with the SPK1 signal peptide for secretion. The recombinant proteins were expressed and purified, then analyzed for antimicrobial activity using plate assay and turbidity reduction assay. In addition, the spent media of the recombinant lactococcal culture was analyzed for its ability to inhibit the growth of the *S. aureus* PS 88.

**Results:**

Extracellular recombinant endolysin (Endo88) and VAPGH (VAH88) was successfully expressed and secreted from *L. lactis* which was able to inhibit *S. aureus* PS 88, as shown by halozone formation on plate assays as well as inhibition of growth in the turbidity reduction assay. Moreover, it was observed that the spent media from *L. lactis* NZ9000 expressing Endo88 and VAH88 reduced the viability of PS 88 by up to 3.5-log reduction with Endo88 being more efficacious than VAH88. In addition, Endo88 was able to lyse all MRSA strains tested and *Staphylococcus epidermidis* but not the other bacteria while VAH88 could only lyse *S. aureus* PS 88.

**Conclusion:**

Recombinant *L. lactis*NZ9000 expressing phage 88 endolysin may be potentially developed into a new antimicrobial agent for the treatment of MDRSA infection.

## Introduction

The rise of antibiotic-resistant bacteria is considered one of the greatest worldwide health challenges to date. This problem is largely contributed by the misuse and overuse of antibiotics. *Staphylococcus aureus*, a Gram-positive opportunistic pathogen is often highlighted due to its clinical significance as it can cause invasive and life-threatening infections such as pneumonia and meningitis (Kulkarni et al., 2019). These infections are becoming a serious threat to global health security as the bacteria have acquired multiple antibiotic resistance mechanisms which render treatment with antibiotics inefficient. Methicillin resistant *S. aureus* (MRSA) and multi-drug resistant *S. aureus* (MDRSA) are now one of the leading causes of severe infections in hospitals (Fujiki et al., 2018). While MRSA is defined by *S. aureus* which is resistant to methicillin, MDRSA is defined as *S. aureus* which is resistant to at least three different classes of antibiotics (Neyra et al., 2014). Hence, alternative antimicrobial approaches are urgently required to combat these infectious diseases.

Bacteriophage treatment, known as phage therapy, has been explored as an alternative to antibiotics (Kakasis & Panitsa, 2019). Bacteriophages are viruses that infect bacteria and reproduce by hijacking the host biosynthetic machinery (Raghu et al., 2012). They only target bacteria cells with high specificity which is a desirable safety feature in therapeutics (Rodríguez-Rubio et al., 2013). While whole phages are desirable due to their ability to replicate and auto-dose, the resistance of bacteria towards phages are also commonly reported (Oechslin, 2018) which is why researchers have extended phage therapy to using just the phage lytic proteins. Phage lysins can be divided into two classes which are virion-associated peptidoglycan hydrolase (VAPGH) and endolysin; both are capable of breaking down the bacterial cell wall (Rodríguez-Rubio et al., 2013). Bacteriophage uses VAPGH to help degrade peptidoglycan during early infection to allow phage genetic material to enter the cell while endolysin is used to lyse the host cell at the end of the lytic cycle, thus releasing new mature phage progenies. So far, there has been no reports of bacteria gaining resistance towards these phage lysins (Kashani et al., 2018a).

Endolysins have been widely studied in the recent decade. Endolysins are peptidoglycan hydrolases that possess a cell wall binding domain (CBD) and one or two catalytic domains. These antimicrobial agents do not have self-replicating characteristics like phages, but they also possess a certain degree of specificity to the bacteria from which they are generated from Denyer et al. (2011). However, they tend to have a slightly broader host range than the phage itself (Schmelcher et al., 2012). Unlike the endolysins, VAPGH is less studied, but some studies have also reported the antimicrobial properties of VAPGH (Borysowski & Górski, 2008). VAPGH also possesses one or two catalytic domains, but it lacks the cell wall binding domain. Thus, both endolysin and VAPGH lytic proteins can be used as antimicrobials when added exogenously to the bacteria due to their capability to degrade peptidoglycan, in particular to Gram-positive bacteria which lacks the outer membrane (Gutiérrez et al., 2018). Rapid lytic action and specificity of both of these lytic proteins make them promising antimicrobial agents.

In this study, *Lactococcus lactis* NZ9000 was used as a host to express endolysin and VAPGH from *Staphylococcus aureus* bacteriophage 88. *L. lactis* has been proven to be a good cell factory for the production of heterologous proteins in many cases due to it’s lack of pathogenicity, simple fermentation requirements, controllable expression using the Nisin Controlled Gene Expression (NICE) system and its Generally Recognized as Safe (GRAS) status (Song et al., 2017). Besides, *L. lactis* can also be used as a live carrier of proteins, especially in the delivery of oral vaccines (Mierau et al., 2005), thus allowing the proteins of interest to be continually produced. To a certain extent, using a live carrier compensates the lack of self-replicating feature of phage lysins.

While *S. aureus* is usually associated with skin diseases, it also causes food poisoning through the production of endotoxins (Argudín, Mendoza & Rodicio, 2010). Therefore, starter cultures with the ability to produce antimicrobials from phages could be beneficial in getting rid of pathogens in food (Kashani et al., 2018a). Also, many cosmetics these days incorporate probiotics (live or cell free extracts) in them due to their potential health benefits (Gueniche et al., 2009), further iterating the potential of *L. lactis* expressing phage lysins against *S. aureus*. Therefore, the main objective of this study is to develop recombinant *L. lactis* NZ9000 secreting phage lysins as antimicrobials against MDRSA. The significance of this study is to enhance the natural beneficial effects of probiotic bacteria against pathogens.

In this study, two lactococcal recombinant strains were developed to examine the ability of phage lysins from Bacteriophage 88 to kill the multi-drug resistant *S. aureus* PS 88 host cells. The full sequence of Phage 88 was obtained from NCBI (GenBank ID: NC_007063.1) and screened for VAPGH (ORF004) and endolysin (ORF006), respectively, based on the domains it possessed and BLAST analysis. ORF006 had a CHAP domain, amidase catalytic site and a SH3 binding domain, typical of many staphylococcal endolysins. ORF004 on the other hand, had a CHAP domain and a glucosaminidase domain, but no cell wall binding domain. While there had been numerous studies on staphylococcal endolysins and to a lesser extent on staphylococcal VAPGH, there are almost no direct comparisons between VAPGH and endolysin from the same phage to date. In addition, there are no reports on VAPGH expressed in lactic acid bacteria thus far and only a handful of studies describing expression of endolysins in lactic acid bacteria.

## Materials & Methods

### Bacterial strains, plasmid and conditions of growth

The plasmid and strains used in this study are listed in Table 1. *L. lactis*, NZ9000 strains were cultured in M17 (Merck) agar and broth (Terzaghi & Sandine, 1975) supplemented with 0.5% (w/v) glucose (GM17) and 7.5 µg/mL chloramphenicol whenever needed. The *L. lactis* NZ9000 strains were incubated statically at 30 °C for 16-18 h. When screening *L. lactis* NZ9000 transformants, M17 agar supplement with 0.5% (w/v) glucose, 0.5 M sucrose and 7.5 µg/mL chloramphenicol was used. *L. lactis* NZ9000 harbouring pNZ8048 was grown in GM17 medium supplemented with chloramphenicol, Cm to a final concentration of 7.5 µg/mL. For the secretion of endolysin (NCBI GenBank Accession number: YP_240699) and VAPGH (NCBI GenBank accession number: YP_240695), SPK1, a signal peptide from *Pediococcus pentosaceus* (Baradaran et al., 2013) was used. Both lysins are from Bacteriophage 88 (ATCC 33742-B1), and were tested primarily against its host strain *S. aureus* subsp. *aureus* PS 88 (ATCC-33742). Top 10 *E. coli* strain (Invitrogen, USA) was grown in LB broth and cultured at 37 °C at 250 rpm. When screening for *E. coli* transformants, LB agar supplemented with ampicillin (100 µg/mL) of 5-bromo-4-chloro-3-indolyl-β-D-galactopyranoside (X-gal) was used. All *Staphylococcus* sp. and *Streptococcus agalactiae* strains were cultured in BHI (Brain Heart Infusion) broth or agar. *S. aureus* and *S. epidermidis* were incubated at 37 °C with agitation at 180 rpm while *S. agalactiae* species were grown at 30 °C without agitation.

**Table 1:** Bacterial strains and plasmids used in this study.

**Bacterial strain and plasmid used**	**Relevant features**	**Sources**
**Strains**		
*L. lactis* NZ9000	Plasmid free strain, with the inserted chromosomal gene *nis* R and *nis* K needed for nisin induction. Host strain for all nisin inducible lactococcal vectors used in this study	Ruyter, Kuipers & De Vos (1996)
*E. coli* TOP10	Cloning host for plasmid pCR-Blunt	Invitrogen
*S. aureus* subsp. aureus PS 88	Clinical isolates from New York City	ATCC 33742
Bacteriophage 88		ATCC 33742-B1
MRSA 6	Clinical isolate	Medical Microbiology Laboratory, UPM
MRSA 7	Clinical isolate	Medical Microbiology Laboratory, UPM
MRSA 8	Clinical isolate	Medical Microbiology Laboratory, UPM
MRSA 10	Clinical isolate	Medical Microbiology Laboratory, UPM
MRSA 12	Clinical isolate	Medical Microbiology Laboratory, UPM
MRSA 20	Clinical isolate	Medical Microbiology Laboratory, UPM
MRSA 22	Clinical isolate	Medical Microbiology Laboratory, UPM
*Staphyloccocus epidermidis*	FDA strain use for food testing	ATCC 1228
*Streptococcus agalactiae* serotype 2	Isolated strain from tilapia fish	Faculty of Agriculture, UPM
*Streptococcus agalactiae* serotype 3	Isolated strain from tilapia fish	Faculty of Agriculture, UPM
**Plasmids**		
pNZ-SPK1-Nuc	Modified pNZ8048 containing PnisA promoter, SPK1 signal peptide with Nuc gene,Cm^r^	Microbial Biotech Laboratory, UPM
pCR-Blunt II- TOPO	∼3.5 kb, Kan^r^, cloning vector	Invitrogen
pNZ8048	∼3.3 kb, nisin-inducible PnisA promoter and Cm^r^	Kuipers et al. (1998)
pNZ-SPK1-VAPGH	∼5.2 kb, Modified pNZ8048 containing PnisA promoter, SPK1 signal peptide fused to a His tagged VAPGH, Cm^r^	This study
pNZ-SPK1-Endolysin	∼4.8 kb, Modified pNZ8048 containing PnisA promoter, downstream His-tagged endolysin gene with SPK1 signal peptide fused to a His-tagged endolysin gene, Cm^r^	This study

**Notes.**

ATCC American Type Culture Collection Kan ^r^ Kanamycin resistance Cm ^r^ Chloramphenicol resistance

### Antibiotic susceptibility of *S. aureus* PS88

The antimicrobial susceptibility test was performed by disc diffusion technique using Mueller-Hinton agar (Merck, Darmstadt, Germany) according to the CLSI guidelines (CLSI , 2016). Antibiotics tested were cefoxitin, penicillin, tetracycline, streptomycin, gentamicin, erythromycin, amoxicillin, vancomycin, kanamycin, meropenem, cefotaxime and chloramphenicol.

### Bioinformatics analysis

The sequence of VAPGH and endolysin (named VAH88 and Endo88, henceforth) were obtained from the GenBank database (accession numbers YP_240695 and YP_240699) for multiple sequence alignment and homology modeling. The amino acid sequences were then compared with other well-studied staphylococcal phage endolysins by MAFFT (Multiple Alignment using Fast Fourier Transform) and the amino acid sequence similarity and identity were determined using Needleman-Wunsch pairwise alignment in EMBL-EBI server (https://www.ebi.ac.uk/Tools/psa/emboss_needle/) (Madeira et al., 2019). Then, the conserved domain was predicted by NCBI Conserved Domain Database (NCBI CDD) and Pfam database. The tertiary structure of the Endo88 was generated by MODELLER 10.1 (Sali & Blundell, 1993; Fiser, Do & Sali, 2000) using a template retrieved from the SWISS-MODEL online server (https://swissmodel.expasy.org/). Twenty models were generated from each run during optimization and 5 models with the lowest Discrete Optimized Protein Energy (DOPE) score was selected for further evaluation (Shen & Sali, 2006). PROCHECK (Laskowski et al., 1993) was used to evaluate the quality of the top 5 models. The tertiary structure of Endo88 was subsequently visualized by the PyMOL Molecular Graphics System, Version 1.2r3pre, Schrödinger, LLC. (https://pymol.org/2/).

### Vector construction

The VAH88 gene of *S. aureus* bacteriophage 88 was amplified using oligonucleotides F-VAH88 and R-VAH88 (Table 2) while the SPK1 signal peptide was amplified using F-SPK1 and R-SPK1. The VAH88 gene was subjected to single digestion with Bam*HI* at the N-terminus and ligated in frame with the signal peptide SPK1, digested with the same restriction enzyme (RE) at the C-terminus to obtain the full fusion gene. The ligation of the fusion gene (SPK1-VAH88) was amplified using F-SPK1 and R-VAH88. The resulting PCR product was subjected to double digestion with *Xba* I and *Pst* I and a His-tag sequence was also incorporated into the reverse primer, R-VAH88 to generate a C-terminal His-tag fusion protein. The fusion protein was cloned into pNZ8048 to generate the recombinant plasmid pNZ-SPK1-VAH88. The electroporation method was used for transformation into *L. lactis* competent cells (Holo & Nes, 1989). Clones containing the ligated plasmids were selected using chloramphenicol. A similar method was applied to amplify and clone the SPK1-Endo88 gene into *L. lactis* NZ9000 competent cells. Both recombinant plasmids were confirmed by standard restriction enzyme digestion and DNA sequencing analysis.

**Table 2:** Primers used for plasmid construction.

Primer name	Primer sequences (5′–3′)
F: SPK1	5′GACGCTGCAGAGATGAAAAAAATATTAACGTTGGTAT-3′
R: SPK1	5′TCACGGATCCAGCATGTACATTCGTCGCAGTT-3′
F: VAH88	5′-GCGCGGATCCATGGGATTACCCAAG-3′
R: VAH88	5′GCGCTCTAGATT[*AGTGGTGATGATGGTGAT]* GTTTATATTTATCTCTTATAAAATAGATACCTTTTAAG-3′
F: ENDO88	5′GCGCGGATCCATGCAAGGCAAAATTAACTAAAAAAG-3′
R: ENDO88	5′GCGCTCTAGATT[*AGTGGTGATGATGGTGAT]* GACTGATTTCTCCCCATAAGTCAC-3′

**Notes.**

Restriction enzymes sites are underlined and italicized. His-tags are italicized and in brackets.

* GGATCC refers to *BamH* I, TCTAGA refers to *Xba* I, CTGCAG refers to *Pst* I.

### Protein expression and purification

For protein expression and secretion of Endo88 and VAH88 by *L. lactis* NZ9000, both intracellular and extracellular protein fractions were analyzed. Overnight culture of recombinant NZ9000 (pNZ-SPK1-VAH88/Endo88) were sub-cultured into fresh GM17 medium and grown to an optical density at 600 nm (OD_600_) of 0.4 and induced with 10 ng/mL nisin A (Sigma Aldrich, USA) for 12 h at 30 °C. Non-induced cells were used as a negative control. Bacterial cells were harvested (4,000× g, 10 min, 4 °C) and the pellet was resuspended in 1X PBS buffer and the cells subjected to sonication using Omni Ruptor 4000 (Omni International, GA, USA) for 1 min by a 30 s pause. This was repeated 10 times. The extracellular protein was extracted and concentrated using 1/10 culture volume of 100% trichloroacetic acid for 2 h. Then, the protein was pelleted down by centrifugation and was washed twice using ice cold acetone, dried and resuspended in 1X PBS. Both intracellular and extracellular proteins were quantified by Bradford assay and subjected to SDS-PAGE and Western blot analysis with mouse anti- His Tag^®^ monoclonal antibody (Novagen, USA). The secondary antibody used was Goat Anti-Mouse IgM Horseradish Peroxidase Conjugated (Thermo Scientific, USA) and detection performed using Advansta Reagent (Matrioux, USA).

For recombinant protein purification, the intracellular and extracellular VAH88 and Endo88 samples were purified, respectively, using complete™ His-Tag Purification Resin following the manufacturer’s instruction (Merck Darmstadt, Germany). For the intracellular protein, the culture of VAH88 and Endo88 were induced, and the cells sonicated and centrifuged as described earlier. Then supernatants were purified using a gravitational dripping method. For the extracellular protein, the recombinant expression cultures of Endo88 and VAH88 were harvested by centrifugation and the supernatants (spent media) were filtered using a 0.22 µm syringe filter. Then supernatants were purified using the same gravitational dripping method as above. The buffer in the final eluted fraction for both intracellular and extracellular of VAPGH and endolysin samples were then exchanged for Tris-HCl (50 Mm NaCl, pH 7.5) using a Pierce™ Protein Concentrator with a polyethersulfone membrane and a 5 K molecular cutoff (Thermo Fisher Scientific, Waltham, MA, USA). The concentration of purified proteins was determined by spectrophotometry using the Bradford assays. Each elution fraction was collected and analyzed by SDS-PAGE analysis.

### Plate assays to assess antimicrobial activities of recombinant VAPGH and endolysin

The lytic activity against *S. aureus* PS 88 cells on agar plate were determined using a method previously described with slight modifications (Delisle, Barcak & Guo, 2006). Plates were prepared as follows: PS 88 was grown in 10 mL of TSB to the mid-log phase (OD_600nm_ 0.4–1.0). Then, the cell culture was harvested by centrifuging at 7,000× g for 10 min at 22 °C. The supernatant was discarded while the pellets were resuspended with one mL of 1X PBS buffer. Next, 1% agarose gel was used in the assay and was prepared by dissolving 1.0 g of agarose powder (Vivantis, Malaysia) in 50 mL of 1X PBS and 50 mL of Tryptic Soy broth (TSB). The mixture was heated using a microwave oven until fully dissolved. After the gel was slightly cooled, 10 *μ*L of the PS 88 was added into the gel and then swirled slowly to mix well. Then, the mixture was poured into three different plates for triplicates and eight small wells were punched into the seeded agar. Finally, 10 µL of intracellular or extracellular protein extract with varying concentrations (1, 5, 10, 25, 50 and 100 µg) were loaded into the wells. Subsequently, the plate was incubated at 37 °C for 24 h. Plates were checked for lysis zones the next day. Ten microlitre of 1X PBS buffer was also spotted onto the plate as negative control while 5 µL chloroform was used as a positive control for halo zone formation. These experiments were performed in triplicates. Plate assays were also performed using 50 ug protein on six different kinds of clinical MRSA strains previously isolated from Hospital Serdang, Selangor, Malaysia (MRSA 6, MRSA 7, MRSA 8, MRSA 12, MRSA 10 and MRSA 20), *Staphylococcus epidermidis*, *Streptococcus agalactiae* serotype 2, *S. agalactiae* serotype 3, *E. coli* and *L. lactis* NZ9000 to test the host range of the recombinants.

### Turbidity reduction assays of PS 88 by recombinant VAH88 and Endo88

To assess the ability of the recombinant VAH88 and Endo88 to lyse PS 88 strains quantitatively, the extracellular purified protein sample was tested in turbidity reduction assays. PS 88 cells were grown to log phase (OD_600_ nm = 0.4–0.6) at 37 °C in Tryptic Soy broth. Next, the culture was centrifuged, and the pellet was resuspended in a reaction buffer consisting of 100 mM Tris-HCl buffer pH 7.5 and the OD_600_ nm was adjusted to 0.75 to normalize the number of cells. To measure lytic activity, 100 µL of each purified protein of extracellular protein sample at the concentration of 50 µg/mL was mixed with cell substrate suspension and the change in OD_600_ nm was recorded every 5 min for 1 h. Untreated PS 88 strain and PS 88 treated with protein from uninduced cells were used as a negative control. These experiments were performed in triplicates. Experiments were carried using a method that was previously described with slight modifications (Kashani et al., 2017b).

### Antimicrobial activity of recombinant *L. lactis* secreting phage lysins

Recombinant *L. lactis* expressing pNZ-SPK1-VAH88 and pNZ-SPK1-Endo88 were grown in media. Next, the cells were pelleted at 4,000× g for 10 min by centrifugation. Spent media (9-mL) was sterilized by filtering through a 0.22 µm filter and neutralized by adding 1.5 mL of 1 M Tris-HCl (pH 9). Mid exponential phase growing PS 88 cells was washed with TSB broth and then resuspended into the same medium. Ten microliters of PS 88 cells were inoculated into the spent media from the earlier lactococcal cultures. The growth of the cells was followed for 12 h at 30 °C, by determining viable counts using TSB agar. These experiments were performed using three biological replicates and three technical replicates each. Experiments were carried using a method previously described with slight modifications (Turner et al., 2007).

### Statistical analysis

The statistical significance for all histograms shown was set at the threshold of *P* < 0.05 and was assessed using the Student’s *t*-test.

## Results

### Antibiotics susceptibility testing of *S. aureus* PS 88

Bacteriophage 88 was purchased from ATCC together with its host *S. aureus* PS 88. According to ATCC, bacteriophage 88’s application is for the assay of MRSA strains. However, upon testing, its host *S. aureus* PS 88, a clinical isolate from New York, was found to be susceptible to cefoxitin (alternative to methicillin). However, it was resistant to multiple other antibiotics including penicillin, tetracycline, streptomycin, gentamicin and erythromycin but was susceptible to amoxicillin, vancomycin, kanamycin, meropenem, cefotaxime and chloramphenicol, other than cefoxitin. Since multi-drug resistant bacteria is defined by resistance to at least three classes of antibiotics (Neyra et al., 2014), PS 88 can be considered a MDRSA. Penicillin is a β-lactam antibiotic, tetracycline is a class of its own, streptomycin and gentamicin are aminoglycosides while erythromycin is a macrolide.

### Bioinformatics analysis

The endolysin of the stapylococcal phage 88 is encoded in ORF006, consisting of 491 amino acids. NCBI CDD classifies Endo88 in Peptidoglycan recognition proteins (PGRPs) and src-homology 3 (SH3_5) domain containing protein. It consists of a Cysteine, Histidine-dependent Amidohydrolase/Peptidase (CHAP) domain at amino acid residues 31–113 (Accession number: pfam05257; *E*-value: 2.96e−08), an Amidase (Ami_2) domain at amino acid residues 198–322 (Accession number: smart00644; *E*-value: 1.05e−24), and a SH3_5 domain at amino acid residues 395-460 (Accession number: pfam08460; *E*-value: 2.03e−21). On the other hand, the VAH88 is encoded in ORF004, consisting of 624 amino acids and classified as a glucosaminidase domain-containing protein. It consists of a CHAP domain at amino acid residues 31-119 (Accession number: pfam05257; *E*-value: 6.09e−05), and a Beta N-acetylglucosaminidase (LytD) domain located at residues 431–624 (Accession number: COG4193; *E*-value: 9.65e−68).

**Figure 1:**
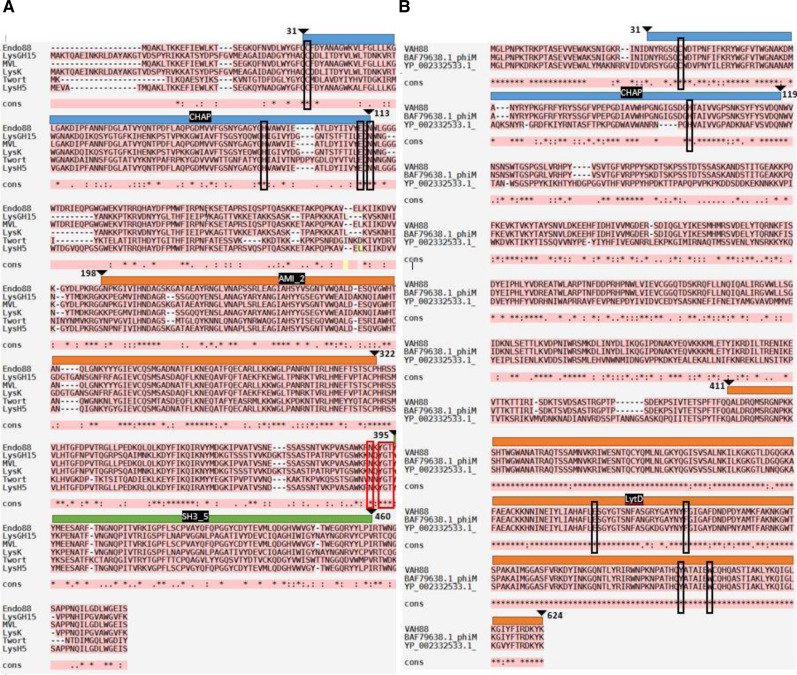
Multiple sequence alignment analysis and prediction of domains of Endo88 and VAH88. Multiple sequence alignment was done by MAFFT. Fully conserved regions are denoted with the symbol (*), conservative substitution regions are denoted with the symbol (:) and semi-conservative substitution regions are denoted signed with symbol. Multiple alignment of Endo88 with LysGH15 (ADG26756.1), MVL (BAF33253.1), LysK (YP_009041293), Twort (AAX92311.1) and LysH5 (ACJ64589.1). Blue, orange and green represent the CHAP, Amidase, and SH3 domains respectively. The conserved region of the catalytic site and peptidoglycan binding site are indicated by black and red boxes respectively (CHAP: C32, H95, E111, N113; SH3: N390, Y392, G393 & T394). (ii) Multiple sequence alignment of VAH88 with HydH5 (BAF79638.1) and YP_002332533.1. Blue and orange represent CHAP and LytD, respectively. The conserved region of the catalytic site are indicated by black boxes (CHAP: C38 & H100; LytD: E509, F528, Y590, W596).

Multiple sequence alignment was performed to compare Endo88 and VAH88 with other well-studied staphylococcal phage lysins (Fig. 1). Endo88 has high amino acid sequence similarity with MVL (BAF33253.1, 99.8%) (Rashel et al., 2007) and LysH5 (YP_002332536.1, similarity: 96.5%) (Rodríguez-Rubio et al., 2012a) but not with LysGH15 (ADG26756.1, 50.6%) (Gu et al., 2011), LysK (YP_009041293, 50.8%) (Becker, Foster-Frey & Donovan, 2008) and Twort (AAX92311,55.3%) (Becker et al., 2015) (Fig. 1A). However, the amino acids forming the catalytic site in CHAP (C32, H95, E111 & N113) and the peptidoglycan binding site (N390, Y392, G393 & T394) are conserved (Gu et al., 2014). On the other hand, VAH88 has high amino acid sequence similarity with another two previously reported VAPGH proteins (BAF79638.1, 99.5%; YP_002332533.1 HydH5, 71.9%) (Rashel et al., 2008; Rodríguez-Rubio et al., 2012b) (Fig. 1B). The LytD domain, including the catalytic site (E509, F528, Y590 and W596) is highly conserved in these VAPGH proteins. The catalytic site for the CHAP domain (C38 and H11) is also highly conserved.

**Figure 2:**
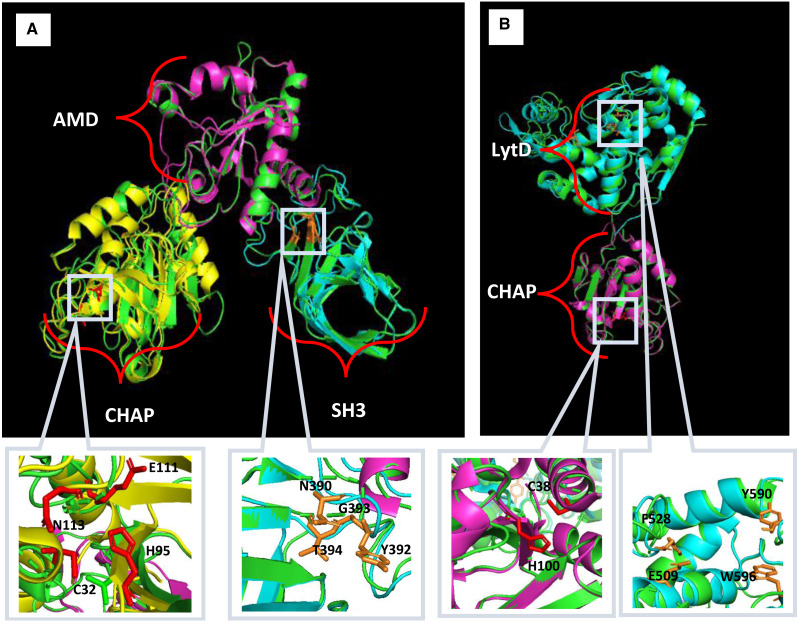
Predicted 3D structure of Endo88 and VAH88 generated by MODELLER and viewed in PyMol. The catalytic site and binding site are shown in grey boxes. (A) Superimposition of Endo88 with respective templates. Green: Endo88; Yellow: 6ist; Purple: 4ols; Cyan: 2mk5. (B) Superimposition of VAH88 with respective templates. Green: VAH88; purple: 6ist; cyan: 6fxp.

Homology modelling for Endo88 and VAH88 was performed using the SWISS-Model online server and MODELLER (Fig. 2). Three templates for each domain were chosen from SWISS-Model. The templates were 6ist.C (Identity: 21.68%) for the CHAP domain, 4ols.A (Identity: 51.22%) for the Amidase domain, and 2mk5.A for the SH3 domain (Identity: 45.08%). The quality of the model was evaluated by Discrete Optimized Protein Energy (DOPE) value and Ramachandran plot. The model with the lowest DOPE value and highest number of amino acids in the most favoured region was chosen for final loop refinement using MODELLER (Fig. S1). For VAH88, templates 6ist.C (Identity: 30.34%) and 6fxo.A (Identity: 42.48%) were retrieved from SWISS-MODEL for the CHAP and LytD domains respectively. The 3D structure of the amino acid residues between the domains were not built because there were no template structures available.

### Cloning of endolysin and VAPGH gene into *L. lactis* NZ9000

The VAH88 and Endo88 genes of *S. aureus* bacteriophage 88 were ligated in frame with SPK1 secretion signal peptide and cloned into pNZ8048 to generate plasmids pNZ-SPK1-VAH88 and pNZ-SPK1-Endo88, respectively (Figs. S2A and S2B). His-tag was placed at the C-terminal of each sequence to facilitate the detection of the expressed proteins. The putative transformants of pNZ-SPK1-VAH88 and pNZ-SPK1-Endo88 were screened by plasmid extraction, single digestion and double digestion analyses on agarose gel electrophoresis (Figs. S2C and S2B). The expected product size for SPK1-VAH88 and SPK1-Endo88 genes were 1,988 bps and 1,557 bps, respectively (Figs. S2C and S2B) inclusive of the restriction enzymes (REs) sites and His-tag sequence. Both genes were successfully cloned into pNZ8048 and were further confirmed by sequencing.

### Expression of SPK1-VAPGH and SPK1-Endolysin from *L. lactis*

**Figure 3:**
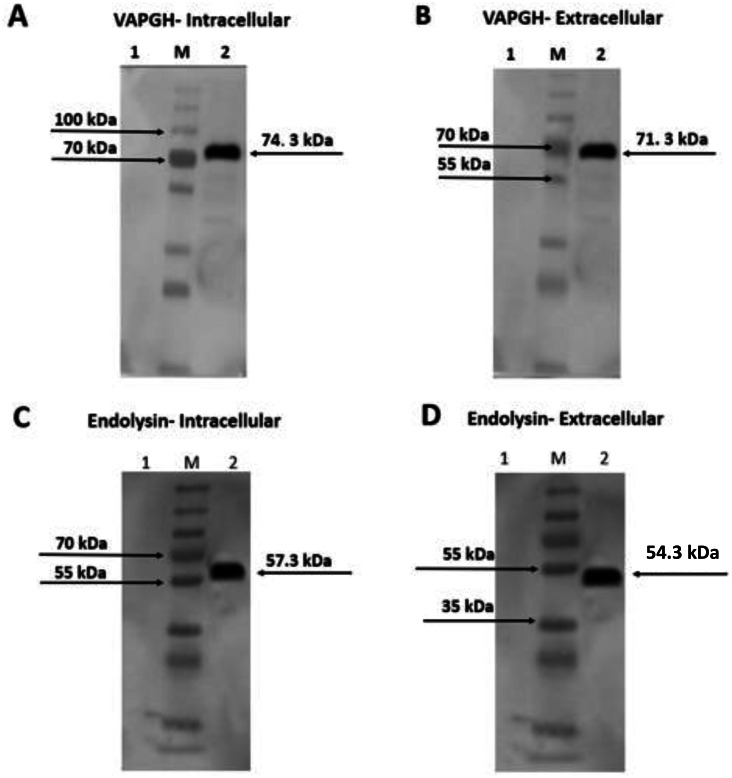
Western blot analysis showing intracellularly and extracellularly expressed VAH88 (A and B) and Endo88 (C and D) by recombinant *L. lactis*. Both lysins were successfully expressed and secreted and the slightly smaller size of the extracellular protein compared to its intracellular counterpart showed that the SPK1 signal peptide was also correctly cleaved. Lane 1: Uninduced, 2: Induced. M: PageRulerTM Plus Prestained Protein Ladder (Thermo Fisher Scientific, USA). The arrow indicates position of the expressed VAH88 and Endo88 recombinant protein.

Intracellular and extracellular crude protein extracts from NZ9000 (pNZ-SPK1-VAH88/Endo88) were analyzed by SDS-PAGE (data not shown) and western blotting (Fig. 3). Bands of expected sizes were seen for both clones. For clones harbouring pNZ-SPK1-VAH88, the expected sizes of intracellular and extracellular protein samples were observed at ∼74.3 kDa and ∼71.3 kDa (Fig. 3A and 3B) respectively, which were not found in the negative controls. For clones harbouring the pNZ-SPK1-Endo88, the expected sizes of intracellular and extracellular protein samples were observed at ∼57.3 kDa and ∼54.3 kDa (Fig. 3C and 3D), respectively, while no bands were expressed in the negative control. The size of the extracellular recombinant VAH88 and Endo88 is smaller compared to the intracellular protein as the SPK1 signal peptide was cleaved from VAPGH and endolysin respectively when it was secreted extracellularly to form the mature protein (Gueniche et al., 2009; Roslan et al., 2020). This analysis showed that both recombinant proteins were successfully expressed and secreted from the recombinant strains.

### Protein purification

**Figure 4:**
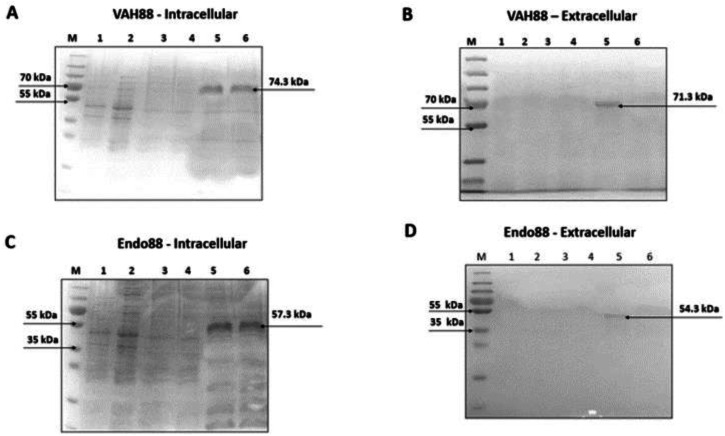
SDS-PAGE analysis showing intracellularly and extracellularly purified VAH88 (A and B) and Endo88 (C and D) by recombinant *L. lactis*. The extracellular protein showed a homogenous single band as there are very few extracellular lactococcal endogenous proteins while residual endogenous proteins were observed in the intracellular fraction. Lane M: PageRulerTM Plus Prestained Protein Ladder (Thermo Fisher Scientific, Waltham, MA, USA). Lane 1: Flow through, Lane 2: Wash 1, Lane 3: Wash 2, Lane 4: Wash 3, Lane 5: Elute 1 and Lane 6: Elute 2. The arrow indicates position of the recombinant purified protein of VAH88 and Endo88.

Purified protein sample of intracellular and extracellular fractions of VAH88 and Endo88 were verified using SDS-PAGE. The detection of intracellular (∼74.3 kDa) and extracellular (∼71.3 kDa) protein band confirmed the purification of VAH88 (Figs. 4A and 4B). For endolysin, the intracellular and extracellular purified proteins were detected at ∼57.3 kDa and ∼54. 3 kDa respectively. Although the intracellular fractions were not purified to homogeneity as other bands were still visible, we did not further purify the protein as the intracellular fraction was not active in the antimicrobial assays described below.

### Plate assay analysis

**Figure 5:**
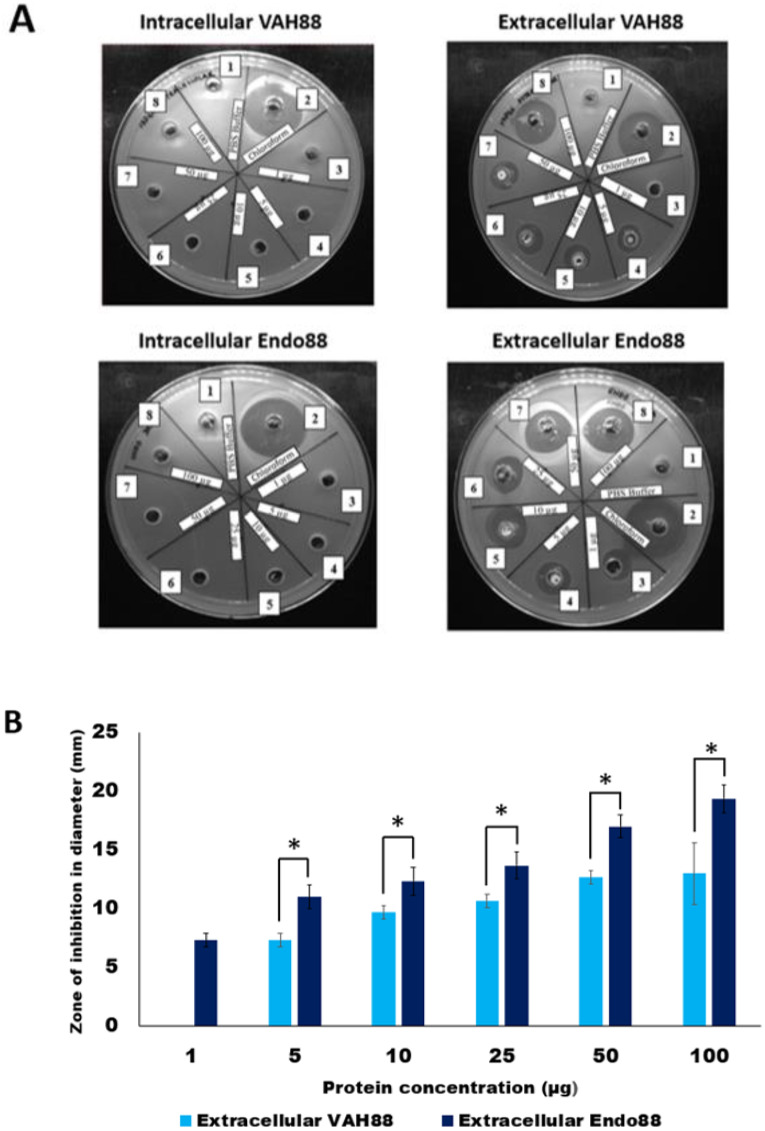
Lytic activity of intracellular and extracellular VAH88 and Endo88 expressed by recombinant *L. lactis* NZ9000. Plate assay showed that only the extracellular fraction was active (A). Well 1: Phosphate Saline Buffer (PBS), Well 2: Chloroform, Well 3: 1 µg, Well 4: 5 µg, Well 5: 10 µg, Well 6: 25 µg, Well 7: 50 µg Well 8: 100 µg. The zone of inhibition for the extracellular fractions indicated that Endo88 was more inhibitory compared to VAH88 (B). Values are the mean of three independent experiments with standard deviation indicated by error bars. An asterisk (*) indicates significance with *p* < 0.05.

The plate assay results (Fig. 5A) demonstrated that the intracellular crude protein of VAH88 did not show any halo zones but the extracellular crude protein of VAH88 exhibited antibacterial lysis activity against PS 88 cells. The same results were shown by Endo88. These results showed that the extracellular protein was functional after the SPK1 signal peptide has been cleaved, leaving the mature protein. The lytic activity appears to be dose-dependent, as the size of halo zone formation was parallel to the increasing amount of proteins. However, the lytic activity of extracellular VAH88 was not observed when using only 1 µg of protein in the well, indicating that the effective dose for VAH88 is higher than endolysin. It was also apparent that Endo88 formed bigger halo zones compared to VAH88 which was confirmed once the zone of inhibition was measured in triplicates (Fig. 5B). Chloroform was used as a positive control in the plate assay to show the inhibitory zone on PS 88 cells while PBS buffer was used as the negative control.

### Turbidity reduction assays

**Figure 6:**
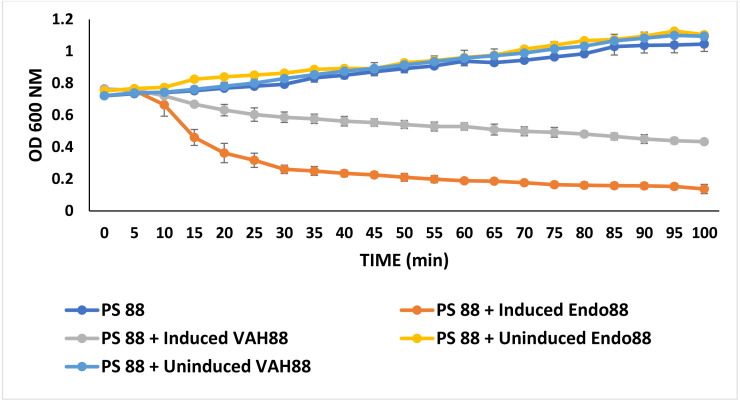
Growth of PS 88 in the presence and absence of recombinant phage lysins. Endo88 inhibited growth of PS88 at a higher rate than VAH88 while the control was not inhibited as expected. Values are the mean of three independent experiments with standard deviation indicated by error bars.

Turbidity reduction assays were performed to quantitatively assess the antimicrobial activities of recombinant *L. lactis* against PS 88. In this experiment, only the extracellular proteins from both strains were tested since there was no inhibition zone observed for intracellular protein in the plate assay experiment. As shown in Fig. 6, both extracellular Endo88 and VAH88 were able to reduce the viability of PS 88 when compared to the control (untreated PS 88 and cells treated with uninduced Endo88 and VAH88). However, Endo88 was able to reduce the viability of PS 88 more compared to VAH88. Therefore, this result further supported the observation from the plate assays that endolysin showed greater inhibition towards PS 88. However, both Endo88 and VAH88 was unable to completely inhibit the growth of PS 88 cells as growth became stagnant after 75 min with no further reduction in OD. Meanwhile, untreated PS 88 and PS 88 treated with uninduced protein fractions continued growing and did not show any inhibition as expected.

### Antimicrobial effect of recombinant *L. lactis* secreting endolysin and VAPGH

**Figure 7:**
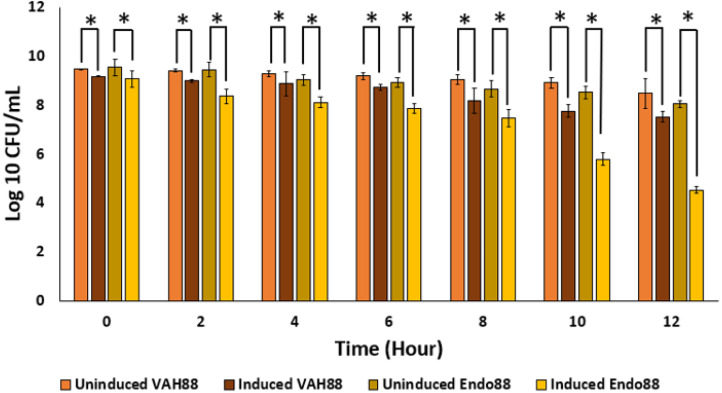
Survival of PS 88 in spent media of *L. lactis* secreting VAH88 and Endo88, respectively. Endo88 was able to reduce growth of PS88 by 3.5 log reductions in 12 h. Values are the mean of three biological replicates and three technical replicates each, with standard deviation indicated by error bars. An asterisk (*) indicates significance with *p* < 0.05.

Since the ultimate aim of the study is to use whole live recombinant lactococcal cells as potential antimicrobials and not the purified proteins, the ability of the recombinant lactococcal cultures to inhibit growth of PS 88 was analyzed. The spent media of both induced and uninduced recombinant strains were inoculated with PS 88 and their growth observed for 12 h. Induced *L. lactis* recombinant strain expressing VAH88 and Endo88 were able to reduce the viability of PS 88 compared to PS 88 in both uninduced recombinant strains until 12 h (Fig. 7). As in the plate assay and turbidity reduction assay, *L. lactis* strain expressing Endo88 was able to significantly reduce the viability of PS 88 better than *L. lactis* expressing VAH88. Overall, treatment with spent media containing *L. lactis* secreted Endo88 resulted in a 3.5-log reduction of PS 88 over 12 h while treatment with spent media containing VAH88 only resulted in a 1-log reduction.

### Lytic activity of crude extracellular endolysin and VAPGH against other bacterial strain

**Figure 8:**
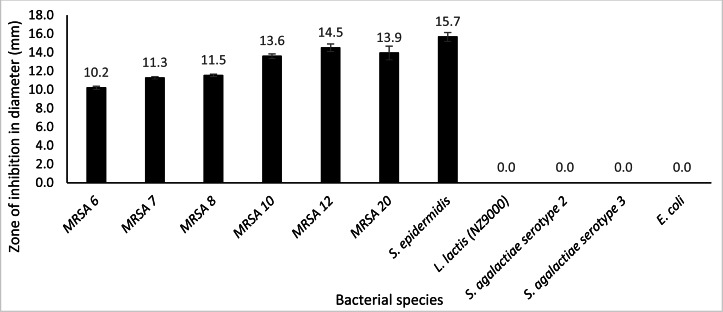
Lytic activity of extracellular protein produced by recombinant clone in plate assay calculated according to the zone size. Endo88 was able to lyse all MRSA strains and *S. epidermidis* but not the other bacteria tested. Values are the mean of three independent experiments with standard deviation indicated by error bars. An asterisk (*) indicates significance with *p* < 0.05.

Plate assay was conducted using six different kinds of clinical MRSA strains (MRSA 6, MRSA 7, MRSA 8, MRSA 12, MRSA 10 and MRSA 20), *S. epidermidis, S. agalactiae* serotype 2, *S. agalactiae* serotype 3, *E. coli* and *L. lactis* NZ9000. From the observation, after incubation for 24 h at 30 °C with 50 µg protein, it was observed that extracellular Endo88 inhibited the growth of all tested MRSA strains as well as *S. epidermidis* which can be observed by the development of halo zone around the wells. Degree of inhibition varied as shown by the difference in zone of inhibition, depending on the strain (Fig. 8). However, none of the zones of inhibition were as big as for its own host, PS 88 which averaged at 17 mm (Fig. 5B). In addition, there were no inhibition zones observed against *S. agalactiae* serotype 2, *S. agalactiae* serotype 3, *E. coli* and *L. lactis.* Inhibition zones were not expected in *L. lactis* NZ9000 since it was used as the host to produce the recombinant phage lysins which did not seem to show any inhibitory effect on the host’s growth. As for VAH88, it did not show any zone of inhibition for all strains tested, inclusive of the other MRSA strains (data not shown) which meant that the VAH88 could only lyse its own host, PS 88.

## Discussion

Based on the above results, two phage lysins from Phage 88 were successfully transformed into *L. lactis* NZ9000, expressed and secreted using the SPK1 signal peptide. Both VAPGH and endolysin do not have a native signal peptide since VAPGH acts from the outside of the cell. On the other hand, endolysin which in nature lyses cells from within is assisted by another protein, holin, which forms holes in the cell membrane, thus allowing endolysin to get to the cell wall (Kashani et al., 2018a).

The recombinant lysins secreted had antimicrobial activity against PS 88, but Endo88 was more efficacious than VAH88 in all analysis studied. VAPGH functions in the initial step of the phage lytic cycle by degrading the peptidoglycan which allows phage infection while endolysin is produced in the lytic cycle during the late phase of the gene and is responsible for the release of the progeny. Therefore, it is understandable that VAPGH is less lytic compared to endolysin since the initial phage infection only involves localized lysis of the cell wall while the release of progenies requires total lysis of the bacteria (Rodríguez-Rubio et al., 2013).

Multiple sequence alignment of Endo88 with other well studied endolysins showed that it was highly similar with MVL and LysH5. MVL was previously reported to possess high lytic ability against Methicillin Resistant *S. aureus* (MRSA), Vancomycin Resistant *S. aureus* (VRSA) and Vancomycin Intermediate-Resistant *S. aureus* (VISA) (Rashel et al., 2007). LysH5 endolysin from *S. aureus* phage vB_SauS-phi-IPLA88 was one of the few endolysins which was also secreted from *L. lactis* to inhibit the growth of MRSA. In their study, they used a signal peptide from a bacteriocin to secrete the endolysin. Unfortunately, most of the protein remained in the intracellular fraction and the endolysin activity of the extracellular fraction was low. Another study expressed LysK, which is a widely studied staphylococcal endolysin, also in *L. lactis* (O’Flaherty et al., 2005). However, in that study, the endolysin was expressed in the intracellular fraction as no signal peptide was used. Lytic efficiency of LysK was comparable to the Phage 88 endolysin in the current study as crude LysK lysates reduced MRSA growth from OD600 nm 0.9–0.6 within 1 h while Phage 88 endolysin reduced the *S. aureus* PS 88 growth from 0.7 to 0.29 in 75 min, although purified protein was used in this study. The sequence of LysK was however, not very similar to Endo88 (∼50% similarity). In another study using a similar strategy, the spent media for another lactic acid bacteria, *Lactobacillus plantarum* secreting Ply115 endolysin reduced growth of *Listeria monocytogenes* by 2.5 log reductions (Turner et al., 2007). To our knowledge, there has been no previous report of VAPGH being expressed in lactic acid bacteria. In fact, there is only very limited studies on VAPGH compared to endolysins, thus we only aligned VAH88 with two other sequences in this study.

In the host range study, Endo88 could lyse all MRSA strains tested besides its host as well as *S. epidermidis* but did not show any lytic activity against *S. agalactiae, E. coli* and *L. lactis* itself. VAH88 on the other hand could only lyse its own host. For endolysins, most reported endolysins can lyse most bacteria within the same species, with many also reported to lyse across species but within the same genus such as *S. aureus* and *Staphylococcus epidermidis* (Kashani et al., 2018a) which is similarly shown in the current study. This is an advantage of using endolysins compared to whole phages which tend to be strain specific, thus needing a cocktail of various phages for it to have any therapeutic use, although broad-spectrum phages have also been reported (Hyman, 2019). Similarly, although not as common, some endolysins can also lyse across the genus (Gilmer et al., 2013). It is probably most desirable that an antimicrobial agent can at least lyse most bacteria within the same species for it to be clinically viable which, fortunately, is a trait most endolysins possess.

There is a notorious difference in protein structure between endolysin and VAPGH. Both VAPGH and endolysins have similar enzymatic active domains that are responsible for peptidoglycan hydrolysis. Yet, in contrast to the endolysin, VAPGH lacks a domain responsible for cell wall binding (Rodríguez-Rubio et al., 2013). The absence of a cell wall binding domain is not surprising given the context in which these proteins function. Receptor binding proteins (RBP) carried in the distal end of the phage tail are responsible for the detection and attachment of bacterial surface receptors. In the current study, it was observed that VAPGH is more specific compared to endolysin in terms of host targets. In a previous study on another staphylococcal VAPGH, HydH5 was also shown to have a limited host range, although it was still able to lyse five out ten *S. aureus* strains and one *S. epidermidis* strain (Rodríguez-Rubio et al., 2012b). However, when the CBD of lysostaphin was fused to HydH5, it was able to lyse all ten *S. aureus* strains and all four *S. epidermidis* strains tested. In addition, lytic activities were also increased. This indicates that having CBD is advantageous to VAPGH. Similarly, most studies showed that deletion of the CBD in endolysins are accompanied with the reduction or loss of lytic activity. Since the sequence of HydH5 is quite similar to VAH88 (∼72%), this same strategy may help increase the host range of VAH88. However, there were also instances where the deletion of a CBD had no effect or even increase the lytic activity of the endolysin (São José, 2018).

There have been many studies of phages and its lysins in various application including food and cosmetics (Gutiérrez et al., 2018; Coffey et al., 2010). Interestingly, the staphylococcal endolysin, LysSA97 was shown to act in synergism with the essential oil-derived carvocrol in the inhibition of *S. aureus* in milk. In addition, the endolysin based topical cream, Staphefekt SA 100 by Micreos is already in the market (Totté, van Doorn & Pasmans, 2017). Although there are limited studies on lactic acid bacteria expressing phage lysins, the concept that probiotics can exert additional antimicrobial properties by producing these antibiotic-like enzymes, termed enzybiotics, is most compelling. Many LAB naturally possesses antimicrobial activity due to the secretion of antimicrobial peptides such as bacteriocin. Therefore, a lactic acid bacterium with the ability to also secrete enzybiotics would significantly increase its antimicrobial efficacy. This is especially desirable when LAB are used as starter cultures in the food industry to keep unwanted microbial cultures at bay. In a study by Gaeng et al. (2000), a lactose-utilizing *L. lactis* strain which can be used as in milk fermentation was used to express phage lysins against *Listeria monocytogenes*. Similarly, an engineered *Lactobacillus casei* BM23 expressing a lactobacilli phage endolysin, Lysdb, was shown to reduce *S. aureus* counts during cheese manufacturing from raw milk (Guo et al., 2016). In addition, many cosmetics and skin care products these days incorporate probiotics into their formulation as probiotics have been shown to confer many health benefits to the skin including preventing and treating allergy, dermatitis, eczema and acne while promoting skin-rejuvenating properties (Lew & Liong, 2013; Roudsari et al., 2015). For example, *Lactobacillus rhamnasus* spent culture supernatant was shown to possess anti-oxidant activities and suppress tyrosinase activity while promoting moisture retention (Tsai et al., 2013). Therefore, topical treatments, such as creams and soaps containing probiotic cells or supernatants, may effectively control staphylococcal skin infection.

## Conclusion

The results of this research demonstrated that both lysins which were encoded by bacteriophage 88 have potential as an antimicrobial agent, with phage endolysins being more effective than VAPGH. Additionally, it was observed that recombinant *L. lactis* NZ9000 cultures secreting phage endolysin themselves could inhibit the growth of MRSA. While *L. lactis* NZ9000 was used in this study as the expression host due to the availability of genetic modification tools, ultimately, it would be ideal to use LAB hosts which have better probiotic properties since all LAB genus, species and even strains give different benefits. In addition, phage lysins can be further genetically engineered to improve its lytic capabilities. The stability of the phage lysins such as in various pH, temperature and salt concentrations can also be analysed further as well as its synergistic effect with other antibiotics. Finally, the effectiveness of the recombinant LAB and its lysins in controlling pathogens in animal models should be tested in further work.

In conclusion, phage therapy including phage lysins may provide a potential alternative to antibiotics, thus helping to alleviate the global antimicrobial resistance problem. In addition, an innovative way of delivering these enzybiotics such as with the use of live lactic acid bacteria cells may confer additional benefits and prospects to a variety of novel applications.

## Supplemental Information

10.7717/peerj.12648/supp-1 Supplemental Information 1 Evaluation of the 3D model structure of Endo88 and VAH88 The quality of the model was evaluated by Discrete Optimized Protein Energy (DOPE) value and Ramachandran plot. The model with the lowest DOPE value and highest number of amino acids in the most favoured region was chosen for final loop refinement using MODELLER

10.7717/peerj.12648/supp-2 Supplemental Information 2 Overview of the construction of pNZ8048 harbouring Endo88 and VAH88 fused with SPK1 signal peptide and His-tag for expression of secretion by *L. lactis* NZ9000 SPK-1-VAH88 and SPK1-Endo88 was successfully cloned into pNZ8048. Figure (A) and (B) shows the schematic diagram of the constructs for VAPGH and endolysin, respectively. Figure (C) and (D) shows analysis of putative positive recombinant plasmid pNZ-SPK1-VAH88 and pNZ-SPK1-Endo88, respectively, by restriction enzyme digestion. Lane M: GeneRuler DNA mix (Thermo Fisher Scientific, USA), Lane 1: Putative positive recombinant plasmids, Lane 2: Single digestion of plasmid using *Xba* I, Lane 3: Double digestion of plasmid using *Pst* I and *Xba* I. Endo88 and VAH88 were successfully cloned into pNZ8048.

10.7717/peerj.12648/supp-3 Supplemental Information 3 Raw Data for Fig. S2 and Figs. 3–4

10.7717/peerj.12648/supp-4 Supplemental Information 4 Raw data for Fig. 5B

10.7717/peerj.12648/supp-5 Supplemental Information 5 Raw data for Fig. 6

10.7717/peerj.12648/supp-6 Supplemental Information 6 Raw data for Fig. 7

10.7717/peerj.12648/supp-7 Supplemental Information 7 Raw Data for Fig. 8

## Additional Information and Declarations

### DNA Deposition

The following information was supplied regarding the deposition of DNA sequences: The sequence for endolysin and VAPGH are available at GenBank: YP_240699 and YP_240695.

### Data Availability

The following information was supplied regarding data availability: All raw measurements are available in the Supplementary Files.
