# Supplementary material for: Lactococcus lactis secreting phage lysins as a potential antimicrobial against multi-drug resistant Staphylococcus aureus
Source: PeerJ. 2022 Mar 1;10:e12648. doi: 10.7717/peerj.12648 (PMC8896023; doi:10.7717/peerj.12648)
Supplement: Supplemental Information 1 — The quality of the model was evaluated by Discrete Optimized Protein Energy (DOPE) value and Ramachandran plot. The model with the lowest DOPE value and highest number of amino acids in the most favoured region was chosen for final loop refinement using MODELLER [file peerj-10-12648-s001.docx]

| **Protein** | **Superimpose with template (Angstrom)** | | | **DOPE** | **Most favoured regions** | **Disallowed regions** |
| --- | --- | --- | --- | --- | --- | --- |
|  | **6ist** | **4ols** | **2mk5** |  |  |  |
| Endo88 | 1.584 | 0.334 | 0.655 | -45100.1 | 83.8% | 1.8% |
|  | **6ist** | **6fxp** | - | -39066.3 | 89 % | 0.3% |
| VAH88 | 0.276 | 0.837 | - |  |  |  |


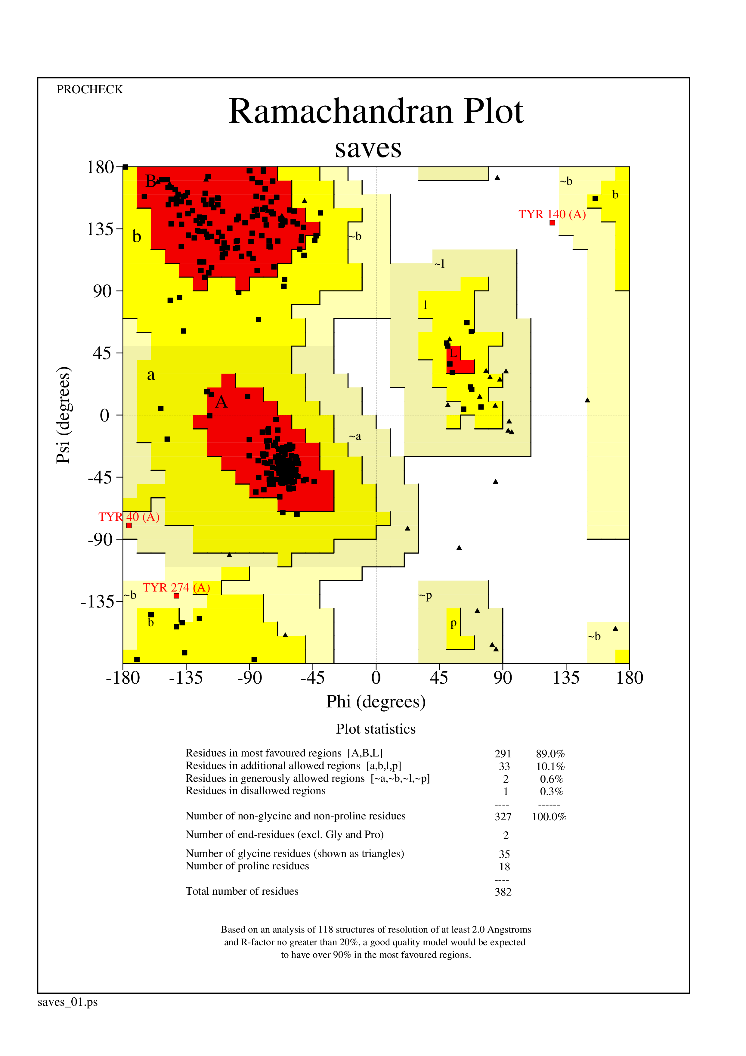


VAH88


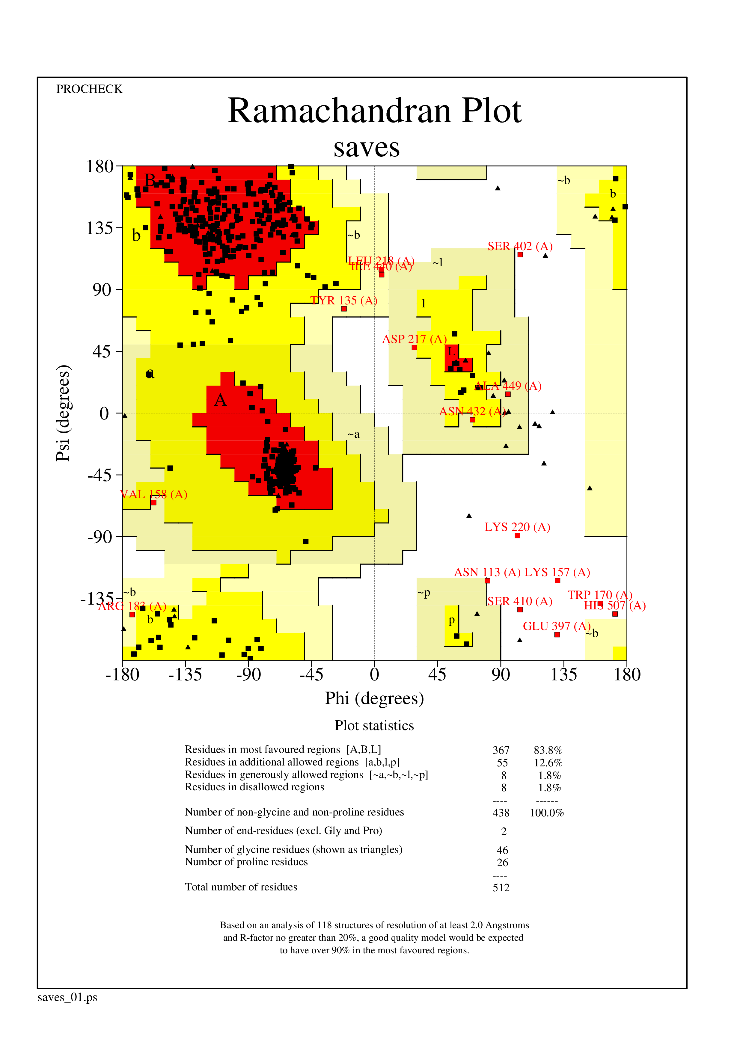


Endo88

**Suppl. Data S1** Evaluation of the 3D model structure of Endo88 and VAH88. The quality of the model was evaluated by Discrete Optimized Protein Energy (DOPE) value and Ramachandran plot. The model with the lowest DOPE value and highest number of amino acids in the most favoured region was chosen for final loop refinement using MODELLER
