# Supplementary material for: Lactococcus lactis secreting phage lysins as a potential antimicrobial against multi-drug resistant Staphylococcus aureus
Source: PeerJ. 2022 Mar 1;10:e12648. doi: 10.7717/peerj.12648 (PMC8896023; doi:10.7717/peerj.12648)
Supplement: Supplemental Information 2 — SPK-1-VAH88 and SPK1-Endo88 was successfully cloned into pNZ8048. Figure (A) and (B) shows the schematic diagram of the constructs for VAPGH and endolysin, respectively. Figure (C) and (D) shows analysis of putative positive recombinant plasmid pNZ-SPK1-VAH88 and pNZ-SPK1-Endo88, respectively, by restriction enzyme digestion. Lane M: GeneRuler DNA mix (Thermo Fisher Scientific, USA), Lane 1: Putative positive recombinant plasmids, Lane 2: Single digestion of plasmid using Xba I, Lane 3: Double digestion of plasmid using Pst I and Xba I. Endo88 and VAH88 were successfully cloned into pNZ8048. [file peerj-10-12648-s002.docx]

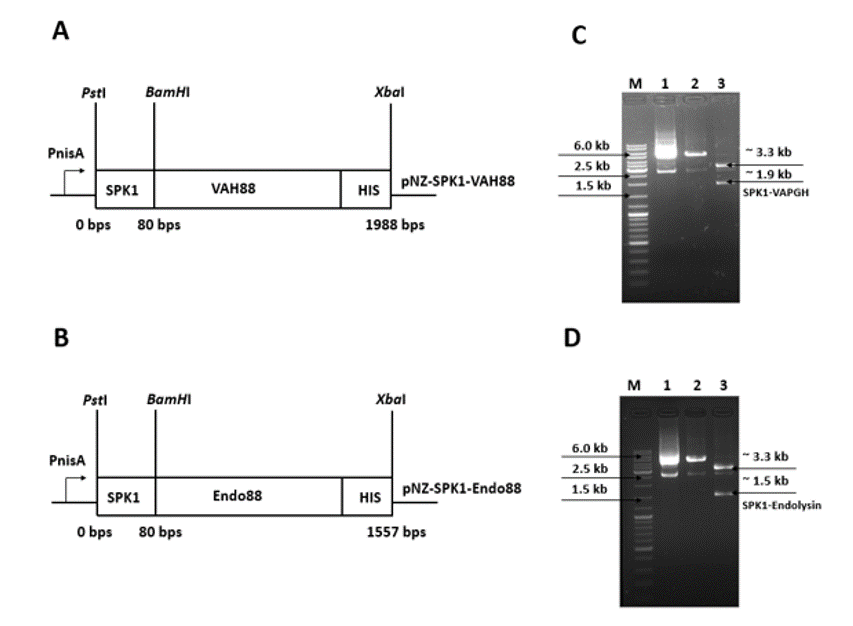


**Suppl. Data S2 Overview of the construction of pNZ8048 harbouring Endo88 and VAH88 fused with SPK1 signal peptide and His-tag for expression of secretion by *L. lactis* NZ9000.** SPK-1-VAH88 and SPK1-Endo88 was successfully cloned into pNZ8048.

Figure (A) and (B) shows the schematic diagram of the constructs for VAPGH and endolysin, respectively. Figure (C) and (D) shows analysis of putative positive recombinant plasmid pNZ-SPK1-VAH88 and pNZ-SPK1-Endo88, respectively, by restriction enzyme digestion. Lane M: GeneRuler DNA mix (Thermo Fisher Scientific, USA), Lane 1: Putative positive recombinant plasmids, Lane 2: Single digestion of plasmid using *Xba*I, Lane 3: Double digestion of plasmid using *Pst*I and *Xba*I. Endo88 and VAH88 were successfully cloned into pNZ8048.
