# Supplementary material for: Lactococcus lactis secreting phage lysins as a potential antimicrobial against multi-drug resistant Staphylococcus aureus
Source: PeerJ. 2022 Mar 1;10:e12648. doi: 10.7717/peerj.12648 (PMC8896023; doi:10.7717/peerj.12648)
Supplement: Supplemental Information 3 [file peerj-10-12648-s003.docx]

**Raw Data for Supplementary Data S2**

**M 1 2 3**


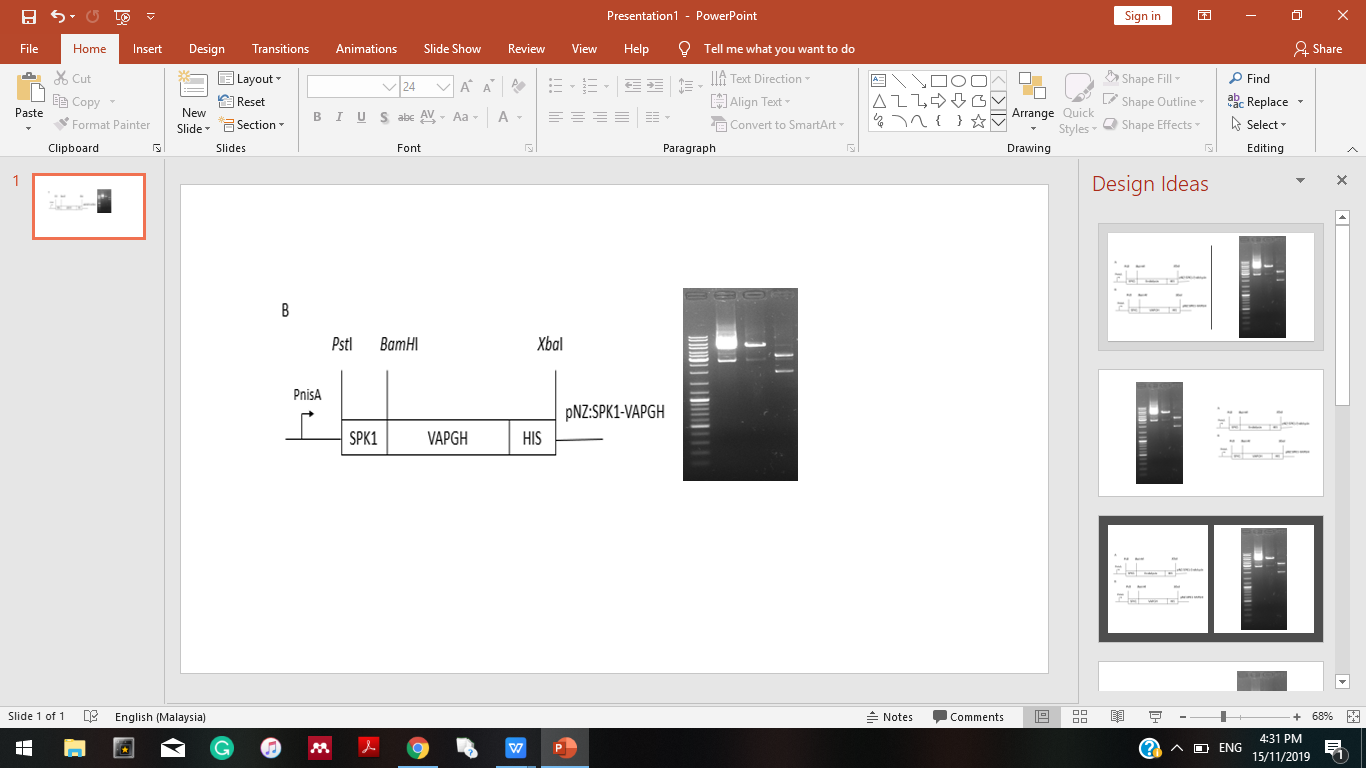


~ 3.3 kb

6000 bps

~ 1.9 kb

3000 bps

1500 bps

**Suppl. Data S2C: Analysis of putative positive recombinant plasmid pNZ-SPK1-VAH88 by plasmid extraction and restriction enzyme digestion**.

Lane M: GeneRuler DNA mix (Thermo Fisher Scientific, USA); Lane 1: Putative positive recombinant plasmids; Lane 2: Single digested putative plasmid using *Pst*I; Lane 3: Double digested of putative transformant using *Pst*I and *Xba*I.


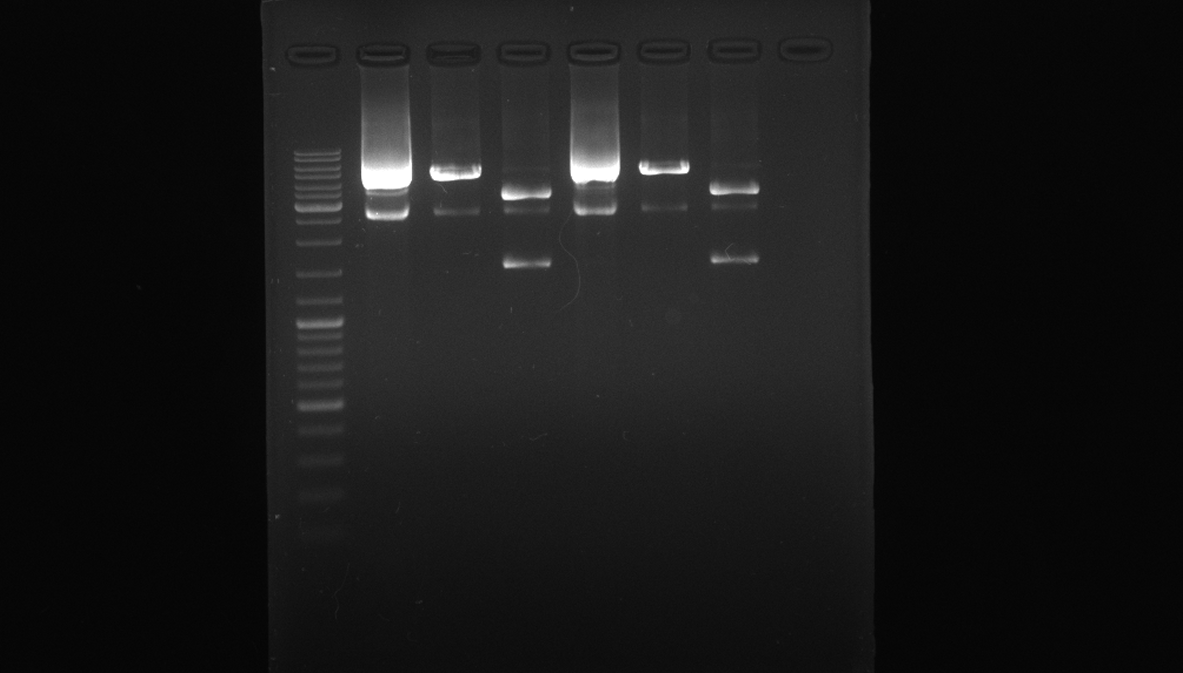


**M 1 2 3 4 5 6**

~ 3.3 kb

6000 bps

~ 1. 5 kb

3000 bps

1500 bps

**Suppl. Data S2D: Analysis of putative positive recombinant plasmid pNZ-SPK1-Endo88 by plasmid extraction and restriction enzyme digestion**.

Lane M: GeneRuler DNA mix (Thermo Fisher Scientific, USA). Lane 1, 4: Putative positive recombinant plasmids; Lane 2, 5: Single digested plasmid of putative transformants using *Pst*I. Lane 3,6: Double digested of putative transformant using *Pst*I and *Xba*I.

**Raw Data for Figure 3-4**

**1 M 2 3 4 5 6 7 8**


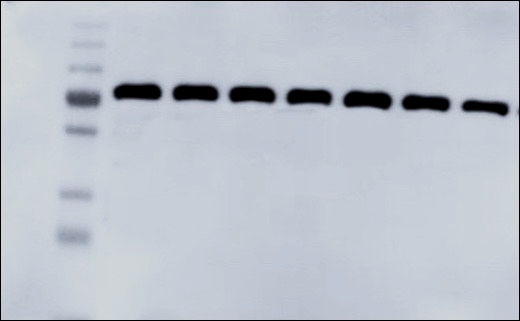


**100 kDA**

**70 kDA**

**74.3 kDA**

**Figure 3A: Intracellular protein expression of clones harbouring the pNZ-SPK1-VAH88 plasmid induced with 10 ng/mL nisin concentration.**

Western Blot analysis. Lane M: PageRuler^TM^ Plus Prestained Protein Ladder (Thermo Fisher Scientific, USA). Lane 2-8: The arrow indicates position of the expressed endolysin recombinant protein.

**1 M 2 3 4 5 6 7 8**


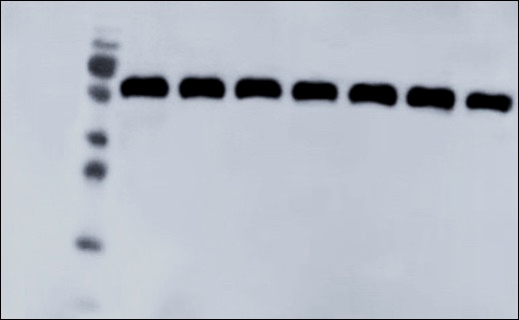


**55 kDa**

**70 kDa**

**71.3 kDa**

**Figure 3B: Extracellular protein expression of clones harbouring the pNZ-SPK1-VAH88 plasmid induced with 10 ng/mL nisin concentration.**

(A) SDS-PAGE analysis (B) Western Blot analysis. Lane M: PageRuler^TM^ Plus Prestained Protein (Thermo Fisher Scientific, USA. Lane 1- 8: The arrow indicates position of the expressed VAPGH recombinant protein.

**
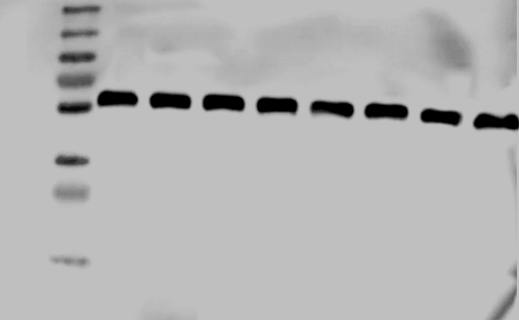
**

**1 M 2 3 4 5 6 7 8 9**

**70 kDa**

**55 kDa**

**57.3 kDa**

**Figure 3C: Intracellular protein expression of clones harbouring the pNZ-SPK1- Endo88 plasmid induced with 10 ng/mL nisin concentration.**

Western Blot analysis. Lane M: PageRuler^TM^ Plus Prestained Protein Ladder (Thermo Fisher Scientific, USA). Lane 1-9: The arrow indicates position of the expressed endolysin recombinant protein.

1 M 2 3 4 5 6 7 8 9

**
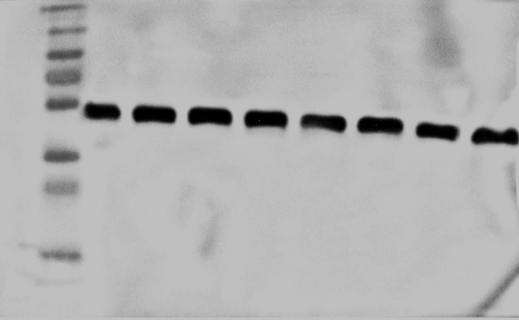
**

**55 kDa**

**35 kDa**

**54.6 kDa**

**Figure 3D: Extracellular protein expression of clones harbouring the pNZ-SPK1-Endo88 plasmid induced with 10 ng/mL nisin concentration.**

Western Blot analysis. Lane M: PageRuler^TM^ Plus Prestained Protein Ladder (Thermo Fisher Scientific, USA). Lane 1-9: The arrow indicates position of the expressed endolysin recombinant protein.

**M 1 2 3 4 5 6**


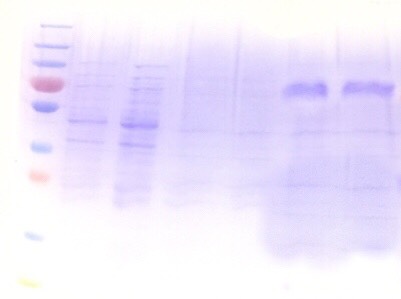


**100 kDa**

**70 kDa**

**74.3 kDa**

**Figure 4A: SDS-PAGE analysis showing intracellularly expressed VAH88 by recombinant *L. lactis***.

Lane M: PageRuler^TM^ Plus Prestained Protein Ladder (Thermo Fisher Scientific, USA). Lane 1: Flow through, Lane 2: Wash 1, Lane 3: Wash 2, Lane 4: Wash 3, Lane 5: Elute 1 and Lane 6: Elute 2. The arrow indicates position of the recombinant purified protein of VAPGH

**M 1 2 3 4 5 6**


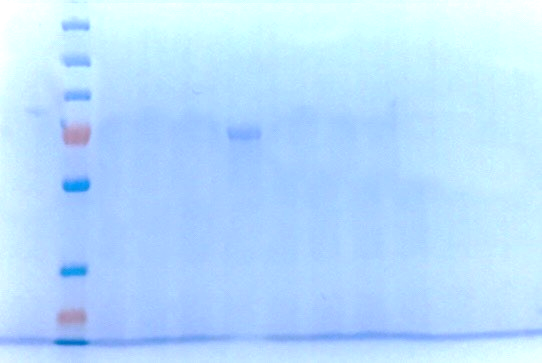


**71.3 kDa**

**70 kDa**

**55 kDa**

**Figure 4B: SDS-PAGE analysis showing extracellularly expressed VAH88 by recombinant *L. lactis***.

Lane M: PageRuler^TM^ Plus Prestained Protein Ladder (Thermo Fisher Scientific, USA). Lane 1: Flow through, Lane 2: Wash 1, Lane 3: Wash 2, Lane 4: Wash 3, Lane 5: Elute 1 and Lane 6: Elute 2. The arrow indicates position of the recombinant purified protein of VAPGH

**M 1 2 3 4 5 6**


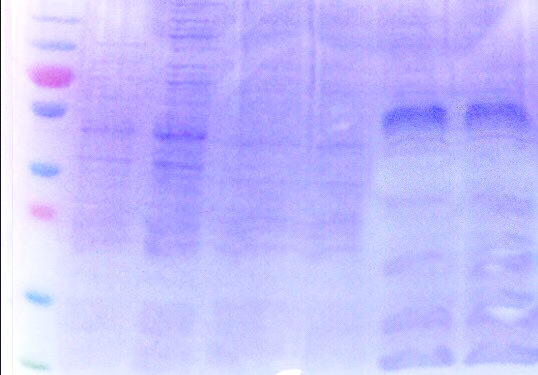


**70 kDa**

**55 kDa**

**57.3 kDa**

**Figure 4C: SDS-PAGE analysis showing intracellularly expressed ndo88 by recombinant *L. lactis***.

Lane M: PageRuler^TM^ Plus Prestained Protein Ladder (Thermo Fisher Scientific, USA). Lane 1: Flow through, Lane 2: Wash 1, Lane 3: Wash 2, Lane 4: Wash 3, Lane 5: Elute 1 and Lane 6: Elute 2. The arrow indicates position of the recombinant purified protein of endolysin.

**M 1 2 3 4 5 6**


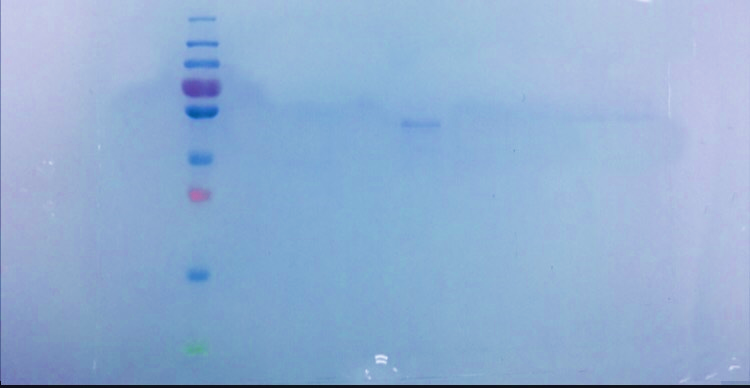


**55 kDa**

**35 kDa**

**54.6 kDa**

**Figure 4D: SDS-PAGE analysis showing extracellularly expressed Endo88 by recombinant *L. lactis***.

Lane M: PageRuler^TM^ Plus Prestained Protein Ladder (Thermo Fisher Scientific, USA). Lane 1: Flow through, Lane 2: Wash 1, Lane 3: Wash 2, Lane 4: Wash 3, Lane 5: Elute 1 and Lane 6: Elute 2. The arrow indicates position of the recombinant purified protein of endolysin.
